# The Components’ Roles in Thermal Stability and Flammability of Cork Powder

**DOI:** 10.3390/ma16103829

**Published:** 2023-05-18

**Authors:** Farnaz Ghonjizade-Samani, Laia Haurie, Ramón Malet, Vera Realinho

**Affiliations:** 1 Poly2 Group, Department of Materials Science and Engineering, Escuela Superior de Ingenierías Industrial, Aeroespacial y Audiovisual (ESEIAAT), Universitat Politècnica de Catalunya (UPC BarcelonaTech), C/de Colom, 11, 08222 Terrassa, Spain; 2 Elix Polymers, Polígono Industrial, Ctra. de Vilaseca—La Pineda s/n, 43110 Tarragona, Spain; 3 GICITED Group, Department of Architectural Technology, Escuela Politécnica Superior de Edificación de Barcelona (EPSEB), Universitat Politècnica de Catalunya (UPC BarcelonaTech), Av. Dr. Marañon 44-50, 08028 Barcelona, Spain

**Keywords:** cork, suberin, lignocellulosic material, thermal stability, flammability

## Abstract

In this study, an analysis of the influence of extractives, suberin and lignocellulosic components on the pyrolysis decomposition and fire reaction mechanisms of a cork oak powder from *Quercus suber* L. is presented. The summative chemical composition of cork powder was determined. Suberin was the main component at 40% of the total weight, followed by 24% of lignin, 19% of polysaccharides and 14% of extractives. The absorbance peaks of cork and its individual components were further analyzed by means of ATR-FTIR spectrometry. Thermogravimetric analysis (TGA) showed that the removal of extractives from cork slightly increased the thermal stability between 200 °C and 300 °C and led to the formation of a more thermally stable residue at the end of the cork decomposition. Moreover, by removing suberin, a shift of the onset decomposition temperature to a lower temperature was noticed, indicating that suberin plays a major role in enhancing the thermal stability of cork. Furthermore, non-polar extractives showed the highest flammability with a peak of heat release rate (pHRR) of 365 W/g analyzed by means of micro-scale combustion calorimetry (MCC). Above 300 °C, the heat release rate (HRR) of suberin was lower than that of polysaccharides or lignin. However, below that temperature it released more flammable gases with a pHRR of 180 W/g, without significant charring ability, contrary to the mentioned components that showed lower HRR due to their prominent condensed mode of action that slowed down the mass and heat transfer processes during the combustion process.

## 1. Introduction

Interest in bio-materials is rapidly growing due to concerns about the environment and their unique characteristics: they are renewable, completely or partially recyclable and bio-degradable [1,2,3].

Cork is a biological tissue that is the outer bark of the oak tree *Quercus suber* L. that acts as a protective layer and is harvested each 9–12 years [4,5,6,7,8]. Due to cork’s remarkable properties such as super compressibility without fracture, full recovery, impermeability and heat insulation, this lignocellulosic material has been widely used in various applications, for example, wine stoppers and construction materials for acoustic and thermal insulation [9,10,11,12].

**Figure 1:**
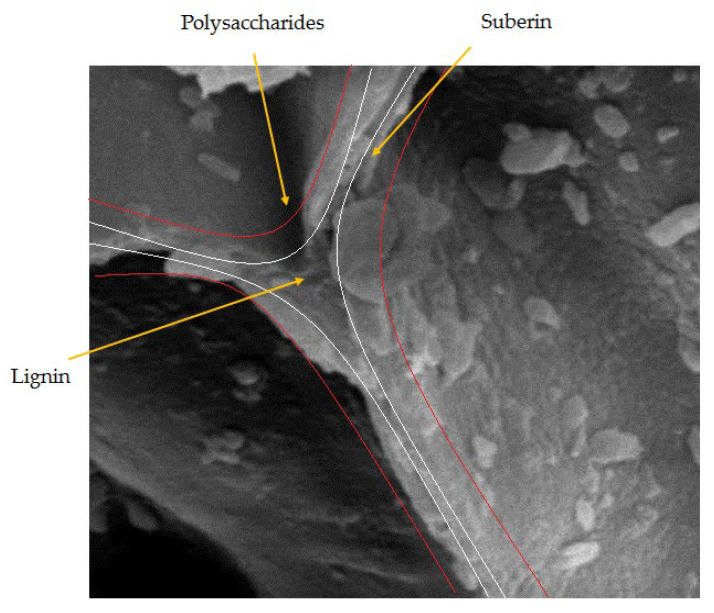
Schematic of cork cells wall presenting the position of each main component.

The macroscopic cellular structure of cork presents an alveolar structure similar to a honeycomb with close cellular structure and thin-walled cells. The cells are rectangular prisms, packed base-to-base in columns parallel to the radial direction of the tree. The walls of cork cells are made of a thin, lignin-rich middle lamella (internal primary wall), a thick secondary wall of suberin and wax lamella and an outer tertiary wall of polysaccharides such as cellulose and hemicellulose (Figure 1) [13,14].

Suberin, the main component of cork, is a polyester consisting of natural aliphatic and aromatic macromolecules linked via ester bonds that provides cork with corrosion resistance and anti-aging characteristics [15]. The second major component of cork is lignin, a highly cross-linked aromatic phenolic polymer which causes durability of cork by increasing stress resistance, mechanical strength and hardness of cork cellular walls. Polysaccharides are also present in cork cellular structure as polysaccharide derivatives consisting of chains of mono-saccharides with intermediate linkages and contribute to the structural rigidity of the cork cells [10,16,17]. Finally, the last parts of cork chemical composition are extractives, which generally include phenolic compounds, terpenoids, fatty acids, resin acids and waxes [18].

Different applications have been reported for components of cork; for example, suberin is used as a starting agent for production of polyurethane [19] and polyester polymers [20,21] and a grafting agent for polymer composites [22], ink additives [23] and cosmetic and pharmaceuticals products [24,25]. Lignin has applications within the sustainable construction industry such as lignin admixture and additives for concrete, lignin modified asphalts, lignin-based paints and coatings [26] or as reinforcing fillers in polymeric composites and an adhesive promoter in natural fiber composites [27,28]. Polysaccharides are used in pharmaceutical [17], food and packaging applications [29,30].

As a result of cork processing and manufacturing of different cork products, the cork industry generates substantial amounts of cork dust (the so-called “cork powder”), approximately 30% of the bulk material, which is usually considered a waste with low economic value and burned in boilers of industrial processes or disposed of in landfills [31,32,33]. Therefore, it is economically interesting to find a more valuable alternative application for this industrial waste bio-material. Recent studies were carried out in order to obtain “green materials” to improve polymers fire retardancy through the development of flame-retardant bio-composites [34]. Lignocellulosic-based materials are capable of producing charred residues, which can be particularly important for developing more sustainable intumescent flame retardants (IFR). Several studies reported that halogen-free flame retardants combined with bio-based materials such as starch [35], lignin [36] and cellulose [37] resulted in an improvement in fire retardancy of thermoplastics by producing a foamed, thermally stable char and consequently protecting the underlying material against fire propagation.

In this sense, it is important to fully understand the chemical nature of cork powder to find a more valuable application for this bio-waste as, for instance, a potential synergistic in flame retardant systems. Although some studies are conducted to investigate the thermal stability of cork powder, to the best of authors’ knowledge, there is a lack of studies regarding its flammability and the mechanisms of action of cork’s main chemical components in its fire reaction.

With all that in mind, the thermal stability and fire behavior of cork powder and of its extractives, the suberin and lignocellulosic components, are investigated in further detail in the present work.

## 2. Materials and Methods

The cork powder was kindly provided by Corcho de Extremadura (Mérida, Spain); dichloromethane, ethanol, methanol, chloroform, sodium methoxide and sulfuric acid were purchased by LabKem (Barcelona, Spain).

The particle size distribution of the cork powder, as received, was determined by a laser diffraction particle size analyzer using a LS 13 320 equipment (Beckman Coulter, Indiana, USA). LS 13 320 software (Beckman Coulter, Indianapolis, IN, USA) was used to calculate the values such as D_50_, which is the median diameter or the medium value of the particle size distribution and the value of the particle diameter at 50% in the cumulative distribution. The D_90_ was also calculated to describe the diameter where 90% of the distribution has a smaller particle size and ten percent has a larger particle size. The mean value, a calculated value similar to the concept of average, was also obtained. An AccuPyc II 1340 helium pycnometer (Micrometritics, Norcross, GA, USA) was used to determine the density of the cork powder. The morphology of the cork particles was analyzed using a JSM-5610 scanning electron microscope (JEOL, Tokyo, Japan). The samples were prepared by sputter depositing a thin layer of gold onto the upper and inner surface of ashes in argon atmosphere using a SCD005 Sputter Coater (Bal-Tec, Los Angeles, CA, USA).

**Figure 2:**
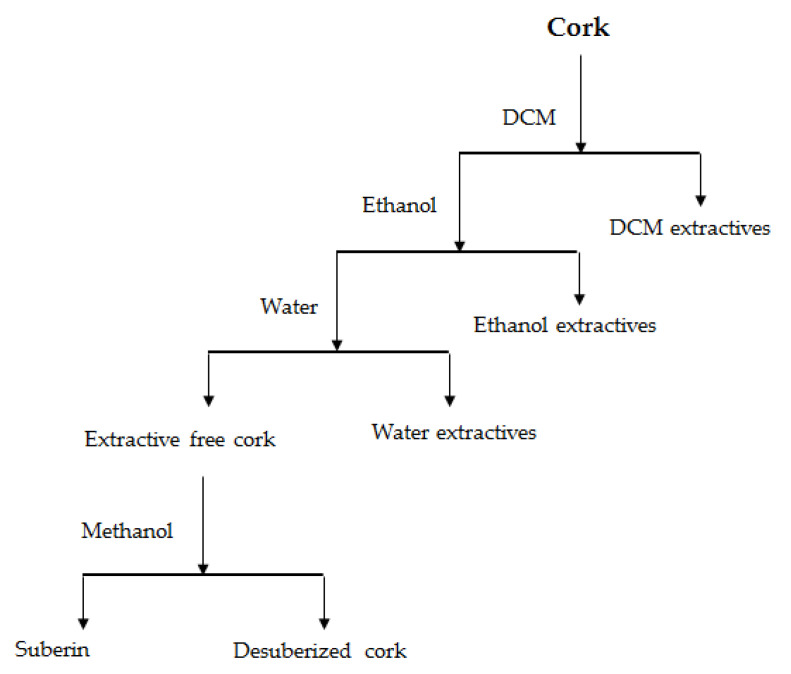
Soxhlet extraction procedure of cork powder.

In order to determine cork composition, chemical analyses that included the determination of ash, extractives, suberin, lignin and cellulose were carried out. All experiments were performed in duplicate. The ash content was determined by incinerating 2 g of cork at 500 °C over 12 h, following TAPPI standard T 15 os-58. Successive Soxhlet extractions (Figure 2) were performed using 3 g of samples to separate extractives with dichloromethane (DCM) for 6 h, ethanol for 8 h and water for 20 h, following TAPPI standards (T204 om-88 and T207 om-93) [38,39]. After each extraction step, the solution was evaporated, and the solid residue weighed. The suberin content of the cork was determined using an extractive-free material by methanolysis for de-polymerization [40]. A total of 1.5 g of extractive-free cork was refluxed with 250 mL of 3% NaOCH_3_ in CH_3_OH over 3 h, filtered and then refluxed with CH_3_OH for 15 min. After filtration, the residue was acidified with 2 M H_2_SO_4_ to pH 6 and evaporated in a rotating evaporator. The residue was suspended in 100 mL of water and extracted with 100 mL CHC1_3_ three times; then the combined extracts were dried over Na_2_SO_4_ salt, filtered, evaporated and weighted as suberin. Lignin was determined after acid hydrolysis with 72% H_2_SO_4_ at 30 °C for 2 h and boiling for 4 h after dilution with water to 4% H_2_SO_4_, following TAPPI T-222 standard. The residue was washed with hot water, dried and determined as lignin [41,42].

Attenuated total reflectance-Fourier transform infrared (ATR-FTIR) spectroscopy using a Nicolet™ 510 (Thermo Fisher Scientific, Waltham, MA, USA) with ZnSe lenses, and a single-reflection diamond ATR element was employed to analyze the chemical nature of cork and its components. The measurements were obtained in the spectral range of 4000 cm^−1^ to 400 cm^−1^.

Thermal stability under pyrolysis conditions was characterized by means of thermogravimetric analysis (TGA) under nitrogen atmosphere, using a STA 449F5 equipment (Netzsch-Gerätebau GmbH, Bayern, Germany) with a constant heating rate of 10 °C min^−1^ from 30 °C to 1000 °C. For each experiment, a mass of 5 mg ± 0.5 mg and a gas flow rate of 250 mL min^−1^ were used.

A micro-scale combustion calorimeter (MCC), also known as a pyrolysis combustion flow calorimeter (PCFC) from Fire Testing Technology (FTT), was used under the procedure of ASTM D7309. In this technique, each sample (5 ± 0.5 mg) was exposed to a heating rate of 1 °C/s from 150 to 700 °C under N_2_. The pyrolysis gases were evacuated into an oven at 900 °C, containing an 80/20 of N_2_/O_2_ mixture, where its total combustion took place. The analysis was performed in triplicate. MCC is a useful instrument to determine the heat release rate and the fuel content of the decomposing volatile products. From this technique, the peak of heat release rate (pHRR), the temperature of the pHRR (T_pHRR_) and total heat release (THR) were measured, and the curves of HRR versus temperature plotted.

## 3. Results and Discussion

### 3.1. Particle Size Distribution, Density and Morphology of Cork Powder

A density of 1.526 g/cm^3^ was registered in accordance with the studies reported for the density of cork powder and cell walls [43,44]. A symmetric distribution with a 218 µm average value of particle size, a D_50_ of 147 µm and a D_90_ of 500 µm for cork powder were obtained.

**Figure 3:**
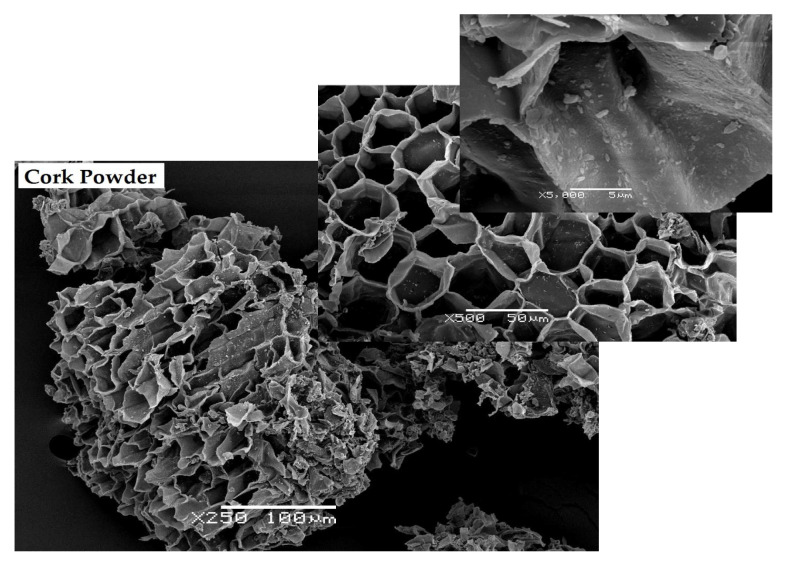
SEM micrographs of cork powder at 250×, 500× and 5000× with a scale bar of 100, 50 and 5 µm, respectively.

Cellular structure of cork powder was observed by scanning electron microscopy (SEM) (Figure 3). The cells are described as rectangular prisms in previous studies and are packed base-to-base in columns parallel to the radial direction of the tree [14]. The anisotropy of cork’s cellular structure, which was observed in SEM, can result in its anisotropic properties [45]. In Figure 3, a representative particle with an average diameter size of 200–250 µm can be observed, in agreement with the obtained average dimensions. Likewise, it was observed that cutting or grinding cork during industrial processing results in the deformation or partial breakage of its cellular structure.

### 3.2. Chemical Composition and Characterization of Cork Components

The results obtained for the summative chemical composition of cork powder are summarized in Table 1. The chemical composition of cork mainly depends on different factors such as geographic origin of cork, climate of the origin, soil, tree dimensions and growth conditions [14]. The results were in agreement with the range reported for cork components in the literature [15,16].

**Figure 4:**
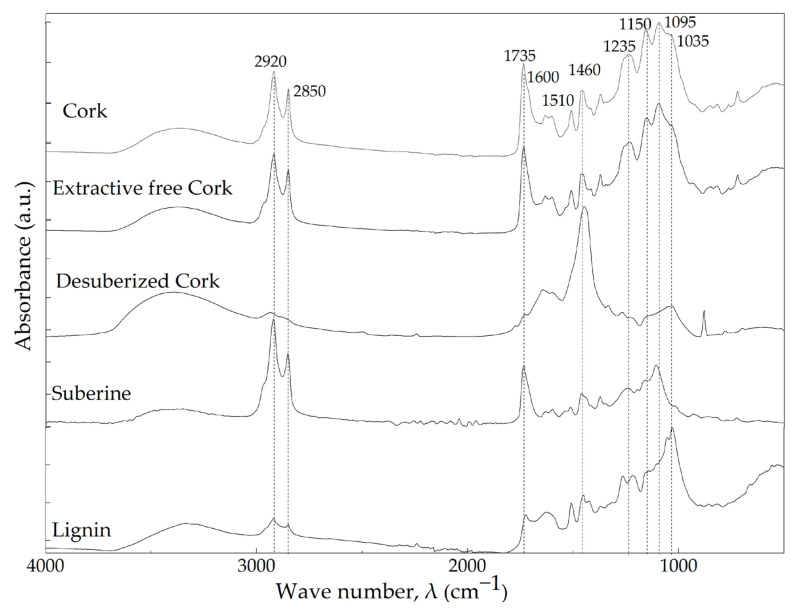
ATR-FTIR spectra of cork powder, extractive-free cork, desuberized cork, suberin and lignin.

The chemical nature of cork powder and its main components was characterized by means of ATR-FTIR spectrometry. Figure 4 shows the normalized ATR-FTIR spectra of cork powder, extractive-free cork, desuberized cork, suberin and lignin.

All materials presented a broad absorbance band between 3700 cm^−1^ and 3000 cm^−1^, characteristic of the hydroxyl groups stretching, presented in their chemical structure. Peaks at 2920 cm^−1^ and 2850 cm^−1^ were mainly attributed to the aliphatic chains of suberin, characteristics of asymmetric and symmetric C–H stretching vibrations, respectively [46,47,48]. The absorbance peaks at 1735 cm^−1^, 1235 cm^−1^ and 1150 cm^−1^ were assigned to C=O, symmetric and asymmetric C–O stretching of the suberin ester group, respectively [46,47,48,49,50,51,52]. The peak registered at 1460 cm^−1^ is also characteristics of C–H asymmetric deformation of suberin, as well as of lignin and polysaccharides [47,50]. Desuberized (lignin and polysaccharides) cork showed a disappearance of the main absorbance peaks of suberin at 2920, 2850, 1735, 1235 and 1150 cm^−1^. This fact indicates that suberin was successfully extracted from the cork powder. Furthermore, besides the mentioned characteristic peaks of suberin, peaks between 1600 cm^−1^ and 1500 cm^−1^ and at 1095 cm^−1^ were registered in the Suberin spectra (see Figure 4), which could be related to lignin and polysaccharide presence. Particularly, C=C stretching registered at 1600 cm^−1^ and 1510 cm^−1^ was assigned to the stretching of G-lignin aromatic ring vibrations [22]. The absorbance peaks at 1095 cm^−1^ and 1035 cm^−1^ are characteristic of C-O vibrations stretching the vibration of polysaccharides and lignin, respectively [47,50,52].

The spectrum of the extractive-free cork was identical to the one of the cork powder, which can be due to the relatively low amount of extractives in the cork (see Table 1).

**Figure 5:**
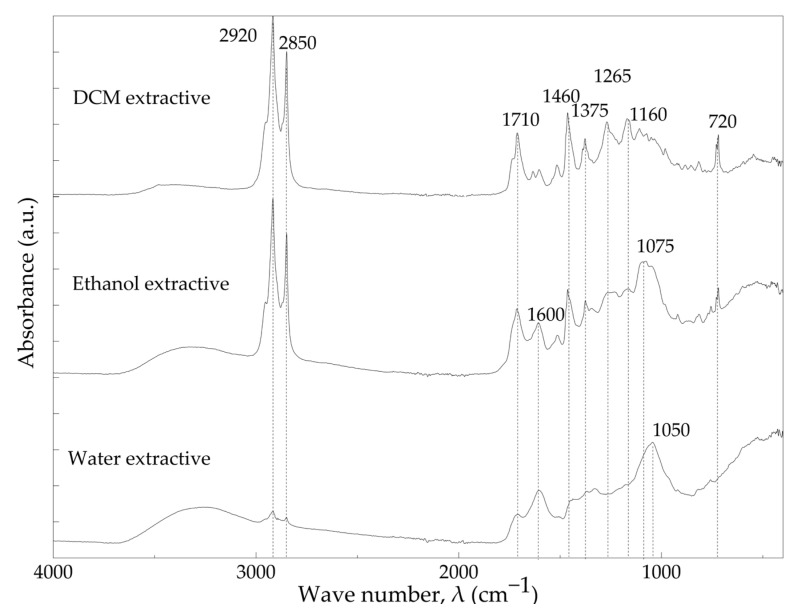
ATR-FTIR spectra of DCM, ethanol and water extractives.

Figure 5 shows the ATR-FTIR spectra of cork extractives. In fact, extractives are not chemically bonded to the cork structure and can be extracted by polar and non-polar solvents [14]. Non-polar extractives were extracted by DCM, and polar extractives were extracted by ethanol and water. Non-polar extractives reportedly consist of waxes with aliphatic and aromatic compounds such as glycerol, fatty acids, triterpenes, while the main parts of polar extractives are phenolic compounds such as phenolic acids and tannins [14,53].

Spectrum of dichloromethane extractives (DCM) showed a broad absorbance band with low intensity between 3700 cm^−1^ and 3000 cm^−1^, which is characteristic of hydroxyl groups. This band has a significantly lower intensity than that of polar extractives. The strong peaks at 2920 cm^−1^ and 2850 cm^−1^ are characteristic of symmetric and asymmetric C–H vibrations present in the aliphatic structure of terpenes of the waxy material. At 1710 cm^−1^, characteristic peaks of carbonyl as carboxylic acid function group and at 1460 cm^−1^ characteristic of aromatic C–C vibration were presented [54,55,56]. In addition, the peak at 720 cm^−1^ was attributed to the C–H bond related to the vinyl group of terpenes [51].

The absorbance band between 3700 cm^−1^ and 3000 cm^−1^ was also observed for ethanol extractives with a higher intensity compared to the non-polar one, corresponding to OH groups of tannins and phenolic compounds. The vibrations at 2920 cm^−1^ and 2850 cm^−1^ are characteristic of C-H vibrations present in aliphatic parts of phenolic acids found in ethanol extractives such as, ferulic acid, vanillic acid and cinnamic acid [14]. The signs at 1710 cm^−1^, 1600 cm^−1^,1460 cm^−1^ and 1075 cm^−1^, respectively, correspond to C=O carboxylic stretching, aromatic C=C stretch, aromatic C–C vibration, C–O asymmetrical stretching, which are typically observed in phenolic compounds [51,56,57].

ATR-FTIR spectra of water extractive of cork also presented the same broad band of OH groups at 3700–3000 cm^−1^. The intensity of the peaks at 2920 cm^−1^ and 2850 cm^−1^, corresponding to C-H vibrations, which are characteristics of phenolic compounds or water-soluble polysaccharides, was much lower compared to the rest of extractives. The signals of C=O stretching at 1710 cm^−1^, aromatic C–C vibration at 1600 cm^−1^ and C–O asymmetrical stretching at 1050 cm^−1^ were also registered for water extractives due to the presence of phenolic compounds [57]. Furthermore, compared to the rest of the extractives, the water extractive showed a decrease of 1710 cm^−1^ and 1460 cm^−1^ peaks intensity and a shift in C–O asymmetrical stretching from 1075 cm^−1^ to 1050 cm^−1^.

### 3.3. Thermal Stability of Cork Powder and Its Main Components

**Figure 6:**
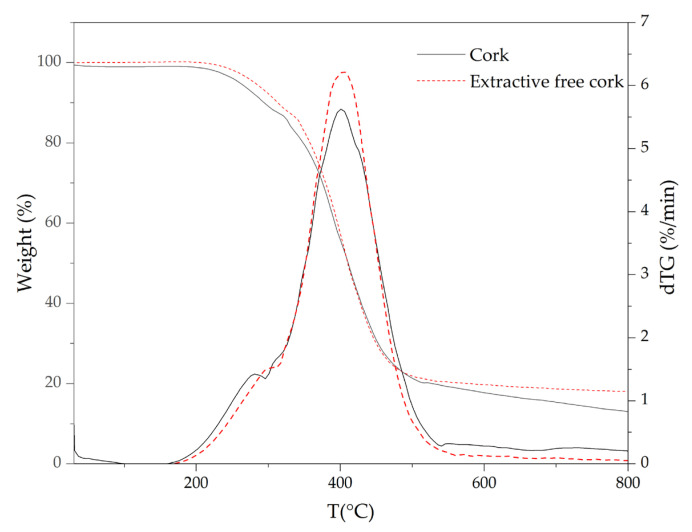
TG and dTG curves of cork and extractive-free cork obtained at 10 °C/min under N_2_ atmosphere.

Thermogravimetric analyses were carried out in order to characterize the role of each component in thermal stability of cork powder. Comparative thermogravimetric curves (TG) of pyrolysis and the first derivative of TG curves (dTG) obtained for cork and extractive-free cork are presented in Figure 6. In addition, the temperature corresponding to the maximum mass loss rate (T_peak_), mass loss (ML) of each decomposition step and the amount of residue remained at 800 °C are shown in Table 2.

Untreated cork started to decompose between 180 °C and 290 °C (see Figure 6), followed by a higher mass loss of 70.1% between 290 °C and 600 °C.

**Figure 7:**
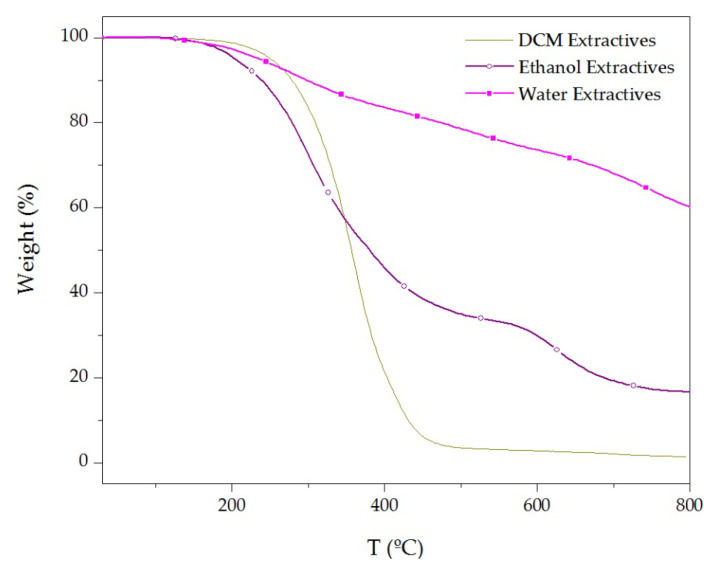
TG curves of cork extractives obtained at 10 °C/min under N_2_ atmosphere.

The removal of extractives from cork shifted the decomposition temperature, at a 5% of mass loss, from 260 °C to 280 °C and led to the formation of a more thermally stable residue between 500 °C and 800 °C. This fact implies that extractives can act as a catalyst by reducing the decomposition temperature of untreated cork and promoting the thermal decomposition of other components. Similar behavior was also observed for the contribution of extractives to the wood thermal degradation [58,59,60]. However, this effect contradicts the role of extractives in thermal stability improvement of cork in oxidative atmosphere [7]. An analysis of the different extractives’ thermal stabilities was also conducted; the TG curves of the extractives are shown in Figure 7.

As it is possible to see in Figure 7, at 5% of mass loss, ethanol extractives showed a lower decomposition temperature compared to DCM and water extractives. Furthermore, above 350 °C, the polar extractives (ethanol and water extractives) showed a lower mass loss rate compared to the one observed for non-polar DCM extractives. In fact, the non-polar extractives only presented a 1.6% residue at 800 °C (see Table 2). Meanwhile, the amount of residue at 800 °C for water and ethanol extractives was 60.1% and 16.7%, respectively.

**Figure 8:**
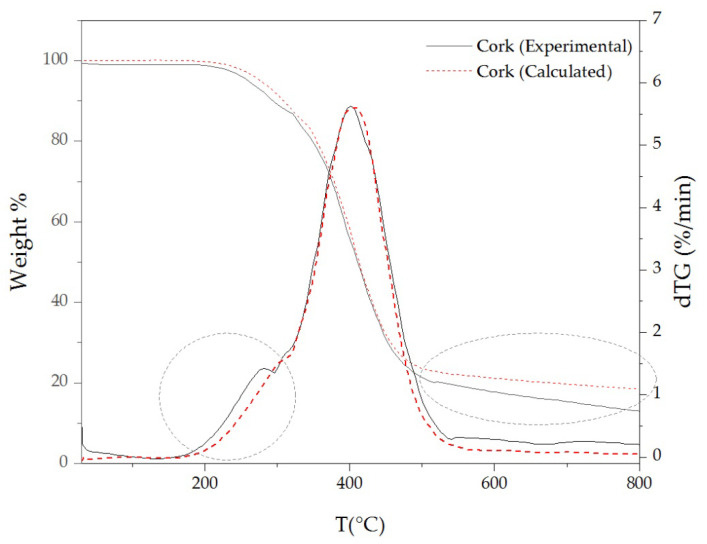
Experimental and calculated TG and dTG of curves of cork at 10 °C/min under N_2_ atmosphere.

In order to evaluate the possible catalytic effect of extractives on the cork thermal decomposition, the experimental and calculated TG and dTG curves of cork were compared (Figure 8). The calculations were obtained from the TG/dTG contribution of the extractive-free cork and individual extractives; calculated cork = (86% × extractive-free cork + 7% × DCM extractives + 3% × ethanol extractives + 4% × water extractives).

The calculated curves show, between 200 °C and 300 °C, a slightly higher thermal stability than those registered in the experimental curves of cork. Thus, when extractives are inherently present in the cork composition, they promote a slightly higher thermal decomposition than expected in that range of temperature. Moreover, between 500 °C and 800 °C, the calculated curve shows a higher weight percentage than that of the experimental curve. These facts reinforce the catalytic effect of extractives on the beginning and end of cork thermal decomposition.

**Figure 9:**
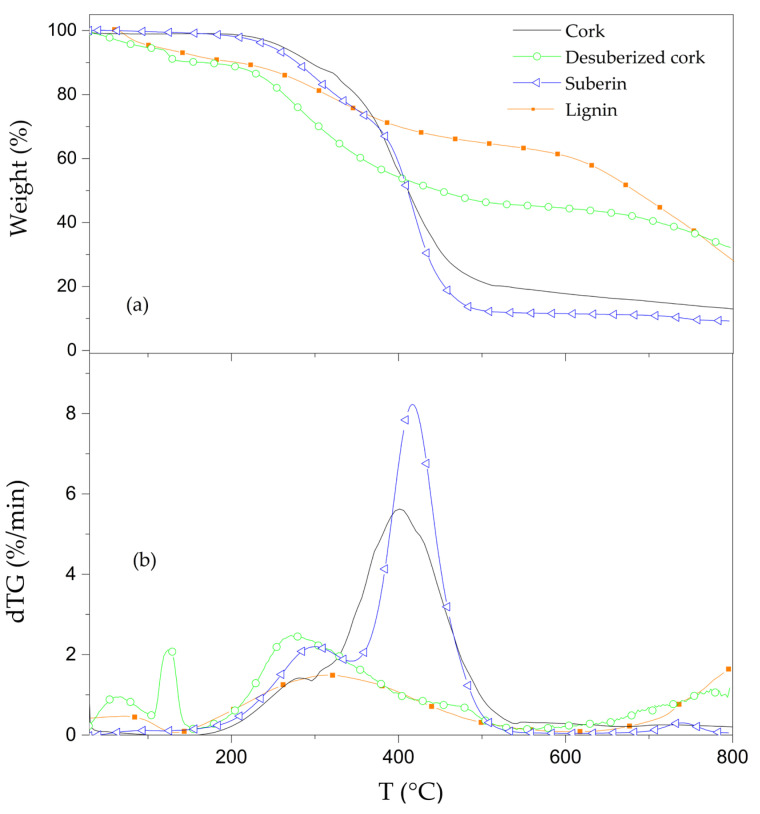
(**a**) TG and (**b**) dTG curves of cork and extractive-free cork obtained at 10 °C/min under N_2_ atmosphere.

The comparative TG and dTG of cork, desuberized cork, suberin and lignin are shown in Figure 9. Desuberized cork (or suberin free cork) showed a lower thermal stability than cork below 400 °C. This might be due to a higher loss of free and bounded water also observed in the case of polysaccharides from different bio-materials [61,62,63]. Its major mass loss occurred at 270 °C, 130 °C lower than that of cork, confirming the thermal stability effect of suberin on cork thermal behavior. The higher residue observed in the desuberized cork samples could be due to the presence of sodium from the sodium methoxide, used for the extraction of suberin [7].

Suberin decomposes in two main steps, similar to cork. The first peak started slowly at 175 °C and continued to 390 °C, with a mass loss of 33.8%. It should be said that the mass loss of this step could be affected by the presence of residual lignin and polysaccharides detected in ATR-FTIR curves. The second and major thermal decomposition step, between 390 °C and 500 °C, showed a 20 °C shift of T_peak_ to higher temperature compared to cork. This indicates that suberin contributes to increase the thermal stability of cork [51]. However, in both the mentioned thermal decomposition steps, the mass loss rate (%/min) was higher than that of cork.

Furthermore, it was reported that suberin is mainly composed of ω-hydroxy acids and α, ω-diacids [41]. The decomposition peaks of ω-hydroxy acids and α, ω-diacids, presented in suberin of different types of cork, were reported to be at 429 °C and 480 °C, respectively [41]. In this sense, it can be concluded that the present suberin is mainly composed of ω-hydroxy acids.

Lignin degraded in a broad step between 140 °C and 600 °C with a mass remaining of 59.4% at 600 °C. At lower temperatures, lignin decomposes due to the cleavage of alkyl-aryl ether linkages, while at higher temperatures the cleavage of aromatic rings and C–C bonds is the cause of thermal degradation [7,41]. A small weight loss below 100 °C, due to free or bonded water, was also observed for lignin. By comparing lignin with desuberized cork (lignin and polysaccharides), it can be seen that lignin appears to be more thermally stable than polysaccharides at temperatures lower that 400 °C. Particularly, from the dTG desuberized and lignin curves, it is possible to observe that the mass loss rate of lignin was lower than that of desuberized sample. In addition, that T_peak_ of the major step decomposition, between 140 °C and 600 °C, was 75 °C higher in the case of lignin. This fact confirms that polysaccharides are the main responsible component for the first temperature peak in the thermal degradation of cork due to its lowest thermal stability among the other main components, and the same results were also observed for the cork components’ contributions to the thermal behavior of cork in oxidative atmosphere [7].

### 3.4. Micro-Scale Combustion Calorimeter (MCC)

In order to assess the flammability properties of cork and its components, a micro-scale combustion calorimeter (MCC) was used. In general, the fire behavior of materials is characterized by the amount of released heat when the material is exposed to a fire. MCC allows to obtain the HRR of materials using a low amount of the sample [64]. As it was described in Section 2, the MCC analysis consists of first heating the sample under pyrolysis conditions in a nitrogen atmosphere; then the degradation gases are purged by an inert gas to the combustor, a chamber at 900 °C with a mixture of nitrogen (80%) and oxygen (20%), where these products are oxidized. The most representative flammability parameters, pHRR (peak heat release rate), T_pHRR_ (temperature to pHRR) and THR (total heat released) are summarized in Table 3. T_pHRR_ represents the maximal heat flow temperature, and THR is obtained by integration of MCC curves.

**Figure 10:**
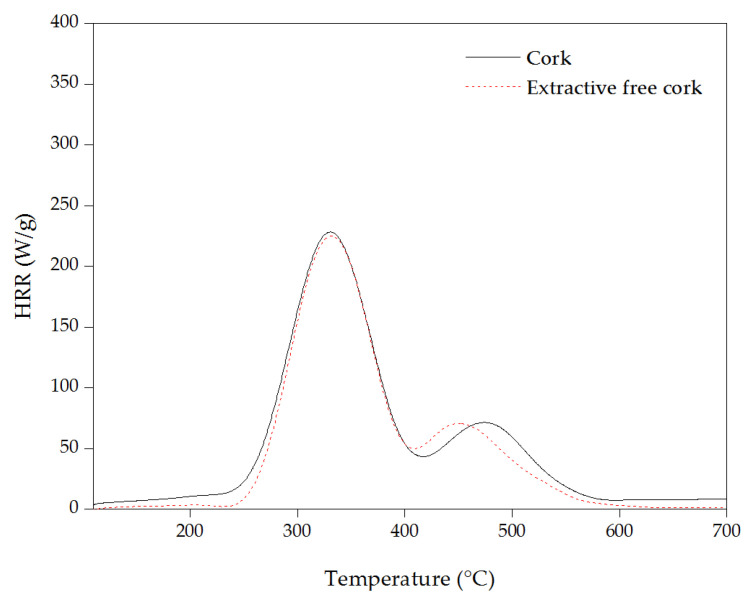
Heat release rate vs. temperature of cork and extractive-free cork.

Figure 10 presents the heat release rate curves of cork powder and extractive-free cork. The cork started to degrade around 200 °C, and the heat release rate continued to increase by increasing the temperature due to the release of combustible gases during decomposition until it reached a maximum of 230 W/g at 330 °C.

The removal of extractives from the cork shifted the starting temperature of degradation from 220 °C to 250 °C with a 12% lower THR, confirming that the extractives act as catalysts by reducing the decomposition temperature of cork and promoting the thermal decomposition of other components; a similar trend was also observed in TGA.

**Figure 11:**
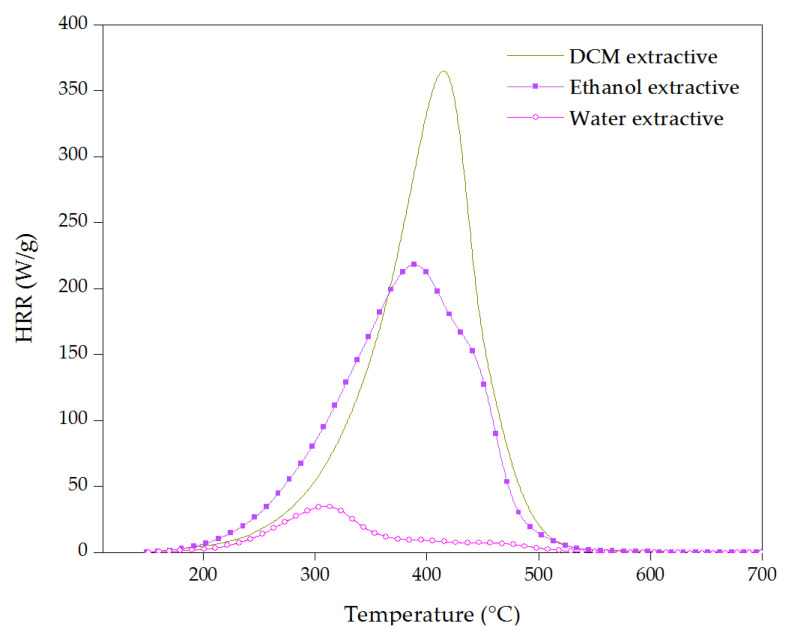
Heat release rate vs. temperature of cork extractives.

Heat release rate curves for cork extractives are presented in Figure 11. The highest value of pHRR, 365 W/g at 410 °C, was registered for dichloromethane extractive. This non-polar extractive started to decompose at 190 °C in one step with almost no residue remaining, showing its high flammability. Ethanol extractive started at 180 °C with a pHRR of 220 W/g at 385 °C, and then water extractive at about 200 °C with a pHRR of 35 W/g at 310 °C, which reached its pHRR at a lower temperature; however, its pHRR and THR were much lower compared to DCM and ethanol extractives. DCM extractives showed the highest T_pHRR_, and together with ethanol extractive, they can be considered the main combustible parts of extractives with the highest pHRR and THR, which can be due to the presence of highly combustible compounds such as waxes, fatty acids and triterpene [53,65].

**Figure 12:**
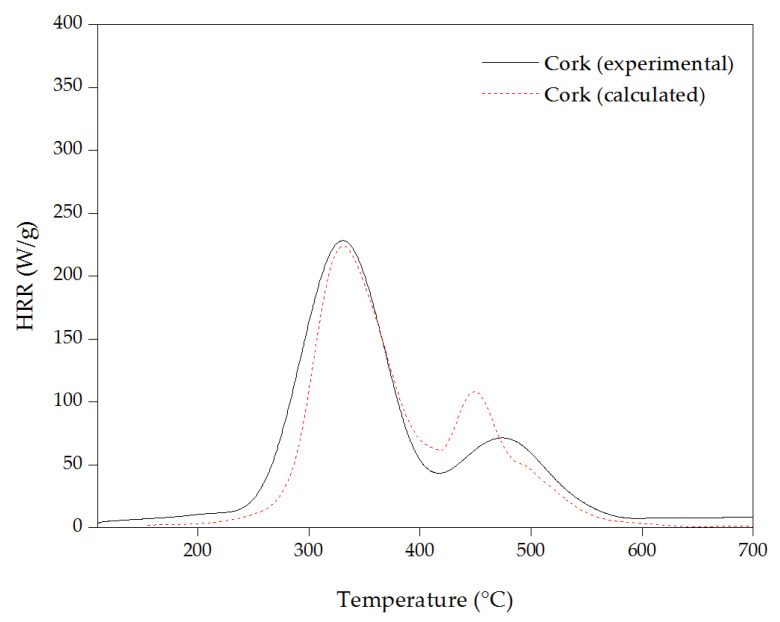
Experimental and calculated heat release rate curves of cork.

In Figure 12, experimental and calculated HRR curves of cork are compared. The calculation was obtained from the HRR contribution of the extractive-free cork and individual extractives, calculated cork = (86% × extractive-free cork + 7% × DCM extractives + 3% × ethanol extractives + 4% × water extractives).

The calculated curve exhibited higher onset decomposition than that obtained in the experimental curve of cork. This observation confirms the catalytic effect hypothesis of extractives, as it was also discussed previously in TGA analysis. Moreover, the second pHRR of cork powder compared to the calculated HRR vs. temperature curve of cork occurred at a higher temperature with a lowest HRR. This fact indicates that, as well as extractives catalyze the beginning of cork thermal decomposition, they also promote the formation of a more thermal-stable carbonaceous residue at higher temperatures (above 350 °C).

**Figure 13:**
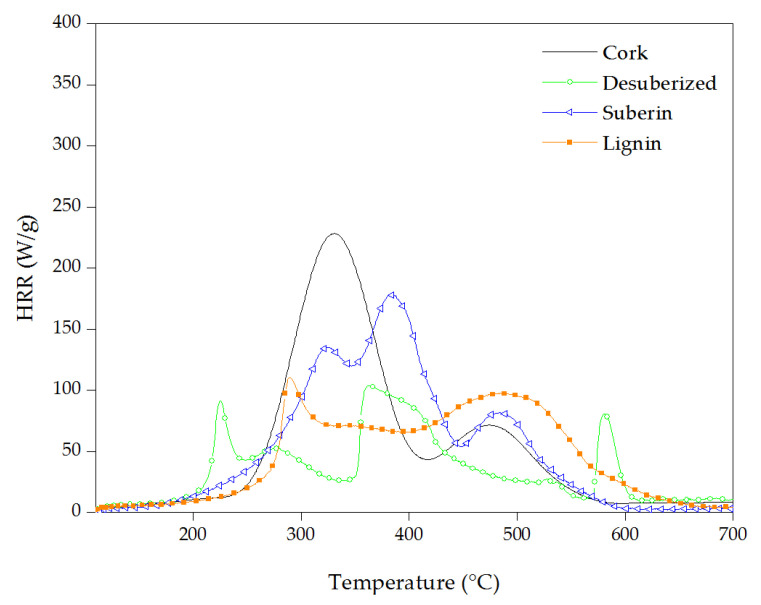
Heat release rate vs. temperature of cork powder and its components.

The comparative HRR curves of cork, desuberized cork, suberin and lignin are shown in Figure 13.

Following the same trend as it was observed in TGA, desuberized cork (or suberin free cork) decomposes at lower temperatures (150 °C) than cork (200 °C). The peaks observed between 200 °C and 320 °C of 90 W/g and 55 W/g, respectively, could be due to the release of polysaccharides of small-chain length [66]. The broad decomposition between 350 °C and 450 °C with a pHRR of 105 W/g could be attributed to the release of combustible volatile products from lignin [67]. The pHRR of 80 W/g at 580 °C is a result of the volatiles’ released combustion when the charred layer formed during lignin pyrolysis was broken [68].

Lignin degraded in two broad steps with a lower T_pHRR_ (290 °C) compared to suberin and cork. By comparing lignin with desuberized cork (lignin and polysaccharides), it can be seen that lignin appears to be more stable than polysaccharides at lower temperatures as it started to decompose at 230 °C, about 50 °C higher than desuberized cork. As it was described by other authors [67,69], the cleavage of the main chain of lignin involves the scission of several oxygen functional groups from its structure with different thermal stabilities. Therefore, a pHRR of 110 W/g at 290 °C that it is followed by a broad area of 70 W/g of HRR until approximately 425 °C can be observed, when probably the char was broken and some flammable gases were released, giving rise to a higher release of heat of 95 W/g at 490 °C.

The MCC curve of suberin shows three steps. The T_pHRR_ of the first peak is at 325 °C. This higher decomposition temperature in comparison with the other constituents of cork is in good agreement with the heat resistant characteristic reported in the literature [70]. The peaks at 390 °C and 480 °C with 175 W/g and 80 W/g of HRR are probably due to the decomposition of the different hydroxy and diacid chains of suberin [7].

## 4. Conclusions

Chemical composition, thermal stability and fire behavior of cork and its main components were investigated in this work. The ATR-FTIR spectra of cork and the individual components showed that suberin was successfully extracted from cork powder; however, the presence of a small fraction of lignin and polysaccharides, in the spectrum of suberin, was detected. Thermal behavior of cork and extractive-free cork, in TGA analysis, revealed that polar and non-polar extractives had a catalytic effect on the thermal degradation of cork powder. It was also observed that by removing suberin from cork, the temperature of maximum mass loss rate shifted to lower temperatures, implying suberin plays an important role in enhancing the thermal stability of cork. Furthermore, MCC analysis was conducted to study the flammability of cork and its components. The removal of extractives from cork shifted the onset temperature of degradation from 220 °C to 250 °C and the temperature of the second pHRR from 475 °C to 450 °C, confirming that extractives act as catalysts at the beginning and end of cork combustion. Among extractives, non-polar extractives are the most combustible ones with the highest pHRR of 365 W/g. It was also concluded that, although suberin is the most heat-resistant component by delaying the thermal decomposition, it exhibits a high flammability as the temperature increases by releasing flammable gases during the combustion without any significant charring effect. It was also noted that the lignocellulosic part of cork starts to degrade by the formation of a char barrier leading to the HRR reduction during the combustion; nevertheless, by increasing the temperature, the char layer was no longer effective and could not protect the underlying material. Further studies of cork powder or its main components as synergistic additives in flame retardant systems can be promising.

## Figures and Tables

**Table 1:** Chemical composition of cork powder.

Chemical Component	Wt% *
Suberin	40
Lignin	24
Polysaccharides	19
Extractives	14
-DCM Extractives	7
-Ethanol Extractives	3
-Water Extractives	4
Ash	3
Total mass	100

* Each value is the average of performed experiments with variation coefficients within 0.02–0.8.

**Table 2:** TG and dTG data of thermal degradation and residue of cork and its main components.

Materials	TD Step	T_peak_ (°C)	ML (%)	R_800°C_ (%)
Cork	1	280	10.5	13.1
	2	400	70.1	
Extractive-free cork	1	300	8.7	17.1
	2	400	71.4	
Desuberized cork	1	65	5.1	32.6
	2	125	4.9	
	3	270	49.8	
Suberin	1	295	33.8	9.5
	2	420	52.1	
	3	740	1.5	
Lignin	1	60	6.7	29.4
	2	350	32.5	
DCM extractives	1	360	95.8	1.4
Ethanol extractives	1	310	66.1	16.7
	2	645	16.9	
Water extractives	1	280	16.3	60.1
	2	520	12.1	
	3	745	11.4	

**Table 3:** Main results obtained from micro-scale combustion calorimetry.

Samples	pHRR (W/g)	THR (kJ/g)	Temperature to pHRR (°C)
Cork	230 ± 12	31.5 ± 0.3	330 ± 1
	70 ± 5		475 ± 1
Extractive-free cork	225 ± 12	27.7 ± 0.9	330 ± 2
	70 ± 5		455 ± 1
Desuberized cork	90 ± 2	24.1 ± 0.4	225 ± 1
	105 ± 8		365 ± 1
	80 ± 4		580 ± 0
Suberin	135 ± 17	27.2 ± 0.5	325 ± 1
	175 ± 11		390 ± 1
	80 ± 2		480 ± 0
Lignin	110 ± 12	26.8 ± 0.5	290 ± 1
	95 ± 12		490 ± 1
DCM extractive	365 ± 4	36.6 ± 0.7	410 ± 2
Ethanol extractive	220 ± 10	30.1 ± 0.9	385 ± 0
Water extractive	35 ± 0	4.1 ± 0.1	310 ± 2

## Data Availability

Not applicable.
